# COMMD1-Deficient Dogs Accumulate Copper in Hepatocytes and Provide a Good Model for Chronic Hepatitis and Fibrosis

**DOI:** 10.1371/journal.pone.0042158

**Published:** 2012-08-06

**Authors:** Robert P. Favier, Bart Spee, Baukje A. Schotanus, Ted S. G. A. M. van den Ingh, Hille Fieten, Bas Brinkhof, Cornelia S. Viebahn, Louis C. Penning, Jan Rothuizen

**Affiliations:** 1 Department of Clinical Sciences of Companion Animals, Faculty of Veterinary Medicine, Utrecht University, Utrecht, The Netherlands; 2 TCCI Consultancy BV, Utrecht, The Netherlands; University of Modena & Reggio Emilia, Italy

## Abstract

New therapeutic concepts developed in rodent models should ideally be evaluated in large animal models prior to human clinical application. COMMD1-deficiency in dogs leads to hepatic copper accumulation and chronic hepatitis representing a Wilson’s disease like phenotype. Detailed understanding of the pathogenesis and time course of this animal model is required to test its feasibility as a large animal model for chronic hepatitis. In addition to mouse models, true longitudinal studies are possible due to the size of these dogs permitting detailed analysis of the sequence of events from initial insult to final cirrhosis. Therefore, liver biopsies were taken each half year from five new born COMMD1-deficient dogs over a period of 42 months. Biopsies were used for H&E, reticulin, and rubeanic acid (copper) staining. Immunohistochemistry was performed on hepatic stellate cell (HSC) activation marker (alpha-smooth muscle actin, α-SMA), proliferation (Ki67), apoptosis (caspase-3), and bile duct and liver progenitor cell (LPC) markers keratin (K) 19 and 7. Quantitative RT-PCR and Western Blots were performed on gene products involved in the regenerative and fibrotic pathways. Maximum copper accumulation was reached at 12 months of age, which coincided with the first signs of hepatitis. HSCs were activated (α-SMA) from 18 months onwards, with increasing reticulin deposition and hepatocytic proliferation in later stages. Hepatitis and caspase-3 activity (first noticed at 18 months) increased over time. Both HGF and TGF-β1 gene expression peaked at 24 months, and thereafter decreased gradually. Both STAT3 and c-MET showed an increased time-dependent activation. Smad2/3 phosphorylation, indicative for fibrogenesis, was present at all time-points. COMMD1-deficient dogs develop chronic liver disease and cirrhosis comparable to human chronic hepatitis, although at much higher pace. Therefore they represent a genetically-defined large animal model to test clinical applicability of new therapeutics developed in rodent models.

## Introduction

Chronic hepatic injury activates a general aetiology-independent process characterized by liver regeneration and fibrogenesis. The sequence of events starts with the primary insult, followed by hepatocyte proliferation and activation of non-parenchymal cells, including hepatic stellate cells (HSCs), liver progenitor cells (LPCs) and macrophages, leading to fibrosis, concomitant failure of regeneration, and finally cirrhosis [1], [2].

HSCs are situated in the space of Disse along the sinusoids. Quiescent HSCs contain vitamin-A loaded lipid droplets. Upon cytokine-induced activation HSCs rapidly loose the lipid droplets, increase the expression of alpha-smooth muscle actin (α-SMA), and secrete extracellular matrix (ECM) components [3]–[5]. HSCs, together with other mesenchymal liver cells and hepatocytes, produce transforming growth factor β (TGF-β) [6]. A transient increase of TGF-β1 in the liver promotes fibrosis with the formation of ECM components, and suppresses hepatocyte proliferation [7]. The hallmark of TGF-β signaling is Smad2/3 phosphorylation [8]. Oppositely, HSCs play an important role in hepatic regeneration as they secrete growth factors including hepatocyte growth factor (HGF). HGF is a potent mitogen for hepatocytes and induces a plethora of other physiological activities comprising anti-apoptosis, morphogenesis and angiogenesis [9], [10]. When mature hepatocytes are extensively damaged or hampered in their replication, the LPCs become activated [11]. LPCs are bipotential and can differentiate both into cholangiocytes and hepatocytes [1], [12], [13].

Chronic liver disease is a worldwide health problem, which in later stages leads to ineffective regeneration, fibrosis, cirrhosis and tumour formation, and can only be solved by organ transplantation. New therapeutic options like anti-fibrotic strategies, or growth factor-mediated interventions are being developed in rodent model studies [2], [14]. The step from *principle-to-practice*, however, remains considerable. The availability of a suitable large animal model with a pathogenesis and pathophysiology of chronic liver disease comparable to that in man is lacking. The availability of such a model would greatly enhance the understanding of possibilities and drawbacks of new clinical interventions. Furthermore it could be tailored to develop effective and safe protocols prior to clinical application [14]. Recently, canine fibrotic liver diseases have been demonstrated to be highly comparable to their human counterparts in both pathophysiological and molecular mechanisms [13], [15]. Comparable pathophysiology itself is not sufficient to become a model animal. A defined etiology allows for more standardized experimental conditions. A dog model with a well-defined genetic aetiology is the Bedlington terrier (BT) which lacks the COMMD1 protein. The absence of the COMMD1 protein in BT, due to a deletion of exon-2 of the *COMMD1* gene, results in a progressive accumulation of copper in hepatocytes leading to chronic hepatitis and cirrhosis [16], [17]. This canine disease was discussed as a potential model for human Wilson’s disease (WD), an autosomal recessive inherited copper storage disorder due to a reduced or absent *ATP7B* gene expression [18], [19], [20], [21]. COMMD1 and ATP7B proteins interact and ATP7B function and stability are impaired upon COMMD1 depletion [22], [23]. These dogs therefore could serve as model for Wilson’s disease and in general for chronic liver disease leading to end-stage liver cirrhosis. Because of their size and life-span, the availability of molecular tools, the possibility and easiness to approach the liver with comparable procedures as performed in man, *e.g.* to take multiple and sequential liver biopsies, combined with a known genetic aetiology these dogs are potentially well-suited for evaluation of new protocols aimed at enhancing liver regeneration and reduction of fibrosis/cirrhosis. A requirement for an accepted model is that the pathogenesis of the disease is known in detail and highly comparable to the human pathology. Therefore we provide a detailed longitudinal analysis of clinical, clinicopathological, and molecular aspects focusing on regenerative and fibrotic pathways of COMMD1-deficient dogs.

## Results

### Physical Examination and Blood Examination

During the entire follow-up period dogs showed no abnormalities at physical examination. Serum ALT was significantly increased at 24, 36, and 42 months of age (2–3-fold), whereas serum AP significantly increased at 18 and 42 months of age. During the 42 months follow-up period, bile acids and albumin remained within reference limits (Table 1).

**Table 1:** Blood examinations.

Parameter&Reference Range	ALT0–54 U/l	AP0–73 U/l	Bile acids0–10 µmol/l	Albumin26–37 g/L
**Age (months)**				
**12**	44(34–75)	73(66–109)	7(3–11)	33(30–34)
**18**	45(35–60)	111 *(91–209)	1(1–2)	30(28–32)
**24**	66 *(43–257)	115(61–120)	7(6–12)	29(27–31)
**30**	83 *(62–153)	87(68–140)	4(1–8)	30(30–32)
**36**	180(69–225)	94(59–153)	2(0–3)	29(29–30)
**42**	112 *(55–201)	101 *(72–219)	2(1–3)	28(27–30)

ALT: alanine aminotransferase; AP: alkaline phosphatase.

Blood examinations during aging in five COMMD1-deficient dogs. Values are expressed as median (range).

* significantly increased serum concentration compared with 12 months of age.

### Histopathological Findings

**Figure 1:**
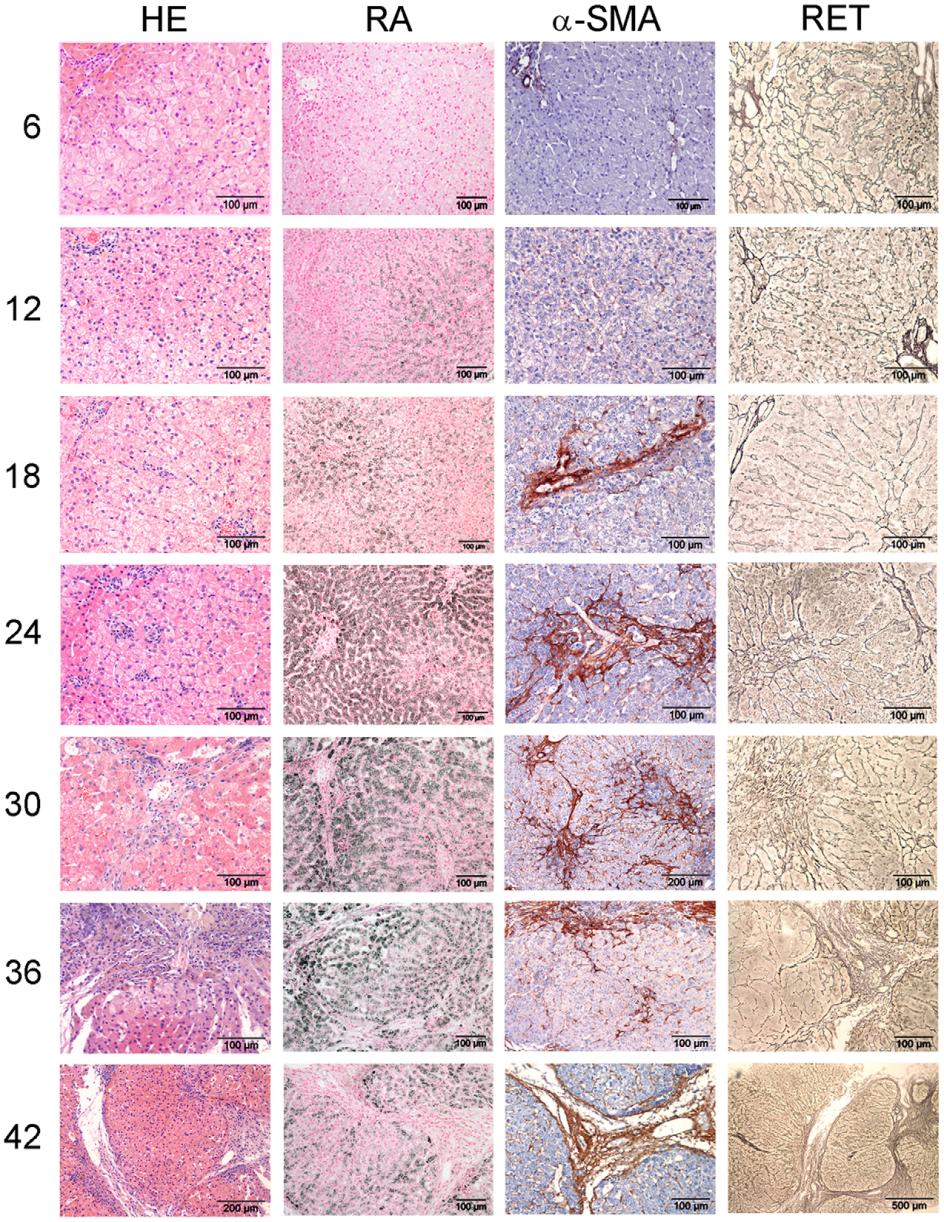
Histological description. COMMD1-deficient dog livers were stained with H&E and RA to assess inflammation and copper accumulation, respectively, and stained for α-SMA and reticulin (collagen type III) to assess fibrosis. Representative pictures of a COMMD1-deficient dog over a period of 42 months are shown. Numbers indicate age in months.

At six months of age all dogs showed a moderate centrilobular hepatic copper accumulation; however no evidence of copper laden Kupffer cells (phagocytosis of copper from destruction of copper laden hepatocytes) or other evidence of hepatitis was observed at this stage (Figure 1). At 12 months of age all animals had extensive copper accumulation diffuse throughout the lobules and in two animals a mild hepatitis was present. At 18 months all dogs showed a slight to mild hepatitis. Although there was some individual variation, the activity of hepatitis progressed to mild and moderate at older age (>24 months) in all dogs.

### Fibrogenesis

α-SMA showed an increasing presence similarly in the COMMD1-deficient dogs and normal age matched controls between 6 (grade 0–1) and 18 months (grade 2). Pathologically increased α-SMA-immunopositivity positivity was seen in all COMMD1-deficient dogs in the centrilobular areas starting from 18 months of age (Figure 1 & Table 2).

**Table 2:** Fibrosis.

Age (months)	6	12	18	24	30	36	42
**α-SMA**grading (0–4)	1(−)	2(1–2)	2(2–2.5)	3(2–4)	3(3–4)	4(2–4)	3(2–4)
p-value	−	0.15	0.048	0.057	0.053	0.054	0.057
**Reticulin**grading (0–3)	0(−)	1(0–1)	0(0–1)	1(0–2.5)	0(0–2.5)	2.5(0–3)	2(1–3)
p-value	−	0.15	1	0.17	0.37	0.17	0.058

Fibrosis scoring: α-SMA (hepatic stellate cell activation) and reticulin grading (collagen type III) in five COMMD1-deficient dogs in a time-dependent copper-induced hepatitis. Data are presented as median (range).

α-SMA grading; 0: no staining of HSCs; 1: a few positive HSCs; 2: diffuse staining of HSCs; 3: mild increased staining of HSCs; 4: marked increased staining of HSC and compared with age matches normal controls.

Reticulin grading; grade 0: normal, grade 1: local mild centrilobular increase, grade 2: multifocal mild to moderate centrilobular increase, grade 3: moderate increase with centro-central bridging or nodular transformation. The uncorrected p-values for comparison of each time point with 6 months of age are reported in the table. No significant differences are present when correcting these p-values for the number of tests.

The reticulin stain showed progressive centrilobular fibrosis starting in one of the five dogs at 24 months of age (Figure 1 & Table 2) and present in all COMMD1-deficient dogs at 42 months of age, finally resulting in centro-central bridging fibrosis and cirrhosis in three of the five animals (at 42 months). Sirius red staining was similar to reticulin staining (data not shown).

### Apoptosis and Regeneration

Active caspase-3 remained undetectable up to 24 months and became visible in the hepatocytes from 30 months of age until 42 months (Table 3). A 2-fold increased median number of Ki67 positive hepatocytes was seen at 36 and 42 months (Table 3), particularly in the three dogs with centro-central bridging fibrosis and cirrhosis, and in the areas with the least copper storage. Both K7 and K19 stainings showed a doubling (median number) of LPCs at 30 months of age and further increase later on, but they showed no differentiation into intermediate cells (Table 3).

**Table 3:** Table **3.** Apoptosis and regeneration.

Age (months)	6	12	18	24	30	36	42
**Casp3**	0(−)	0(−)	0(−)	0(−)	0.1(0–3)	0.1(0–1.1)	0.1(0–0.1)
p-value	−	NA	NA	NA	0.15	0.17	0.35
**Ki67**	0.9(0.6–2.9)	0.8(0.4–1.1)	1.1(0.7–2.0)	0.5(0.3–1.3)	0.7(0.1–1.0)	2.3(0.1–4.4)	2.1(1–4.6)
p-value	−	0.79	0.88	0.25	0.13	0.88	0.5
**K19**	1.9(1.2–3.2)	2.5(1–3.3)	3.2(2.7–3.4)	1.8(1.5–3.3)	3.7(2.5–5.3)	3.6(2.8–7.4)	5.2(4–9.6)
p-value	−	0.10	0.063	0.58	0.058	0.063	0.125

NA: not available.

Number of (active) caspase-3 (Casp3) positive hepatocytes per 10-times objective (manual count), number of Ki67 positive hepatocytes per 10-times objective (manual count), and number of K19 positive cells in the limiting plate per portal area in five COMMD1-deficient dogs in a time-dependent copper-induced hepatitis. Data are presented as median (range).

The uncorrected p-values for comparison of each time point with 6 months of age are reported in the table. No significant differences are present when correcting these p-values for the number of tests.

### Gene-expression Profiling

**Figure 2:**
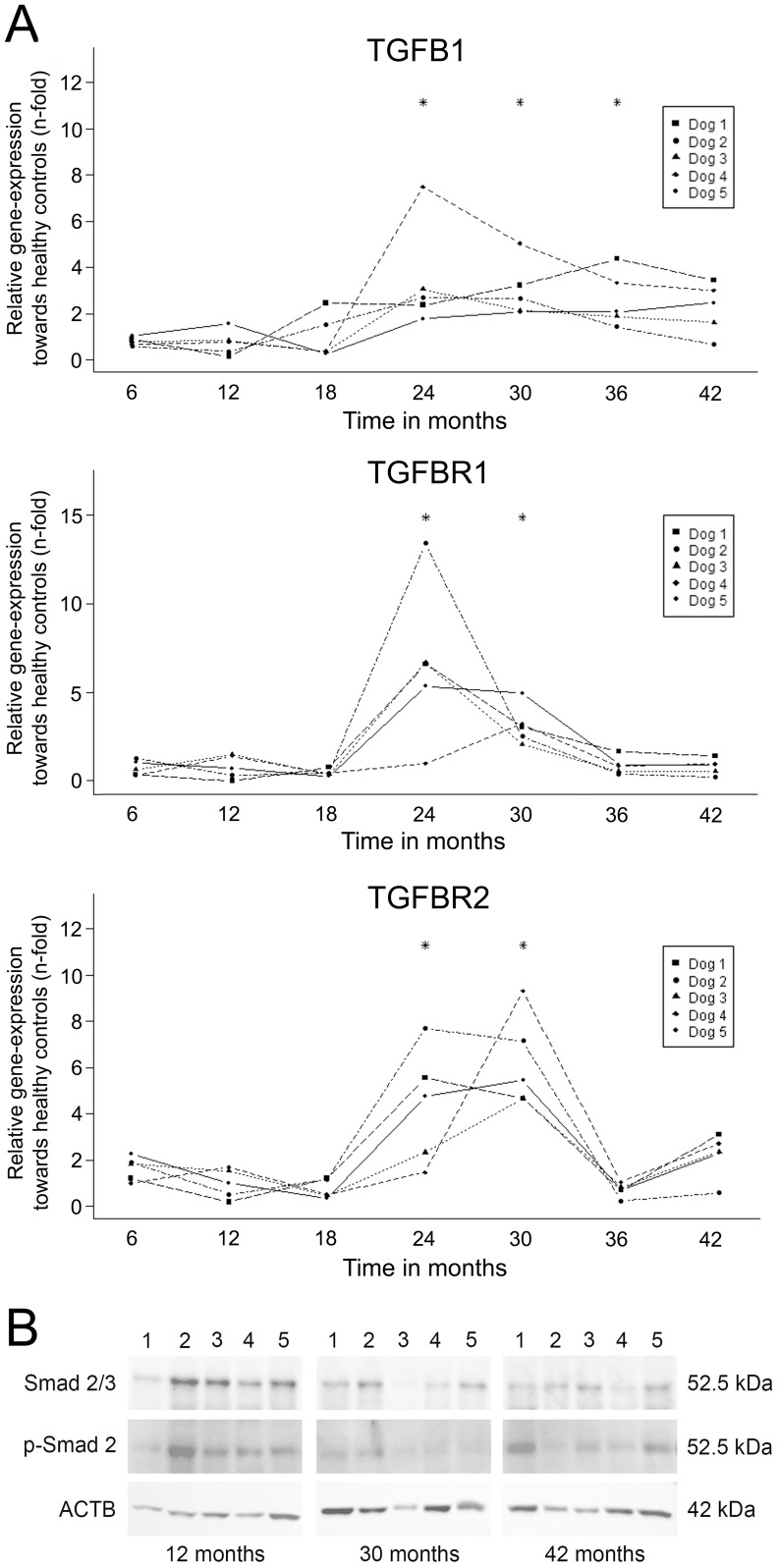
TGF-β1 pathway. (**A**) Gene-expression profiling. Interaction plots of Q-PCR data of important mediators of fibrogenesis in a time-dependent copper-induced hepatitis in five COMMD1-deficient dogs. Q-PCR results were normalized against the expression of six control dogs (one to three years of age). Linear mixed-effect modeling was used; * significant difference corrected for multiple testing. (**B**) Western blot analysis on the activation of TGF-beta signalling (total Smad2 and phosphorylated Smad2 (Ser727)) of five COMMD1-deficient dogs at 12, 30, and 42 months. β-actin (ACTB) served as loading control.

**Figure 3:**
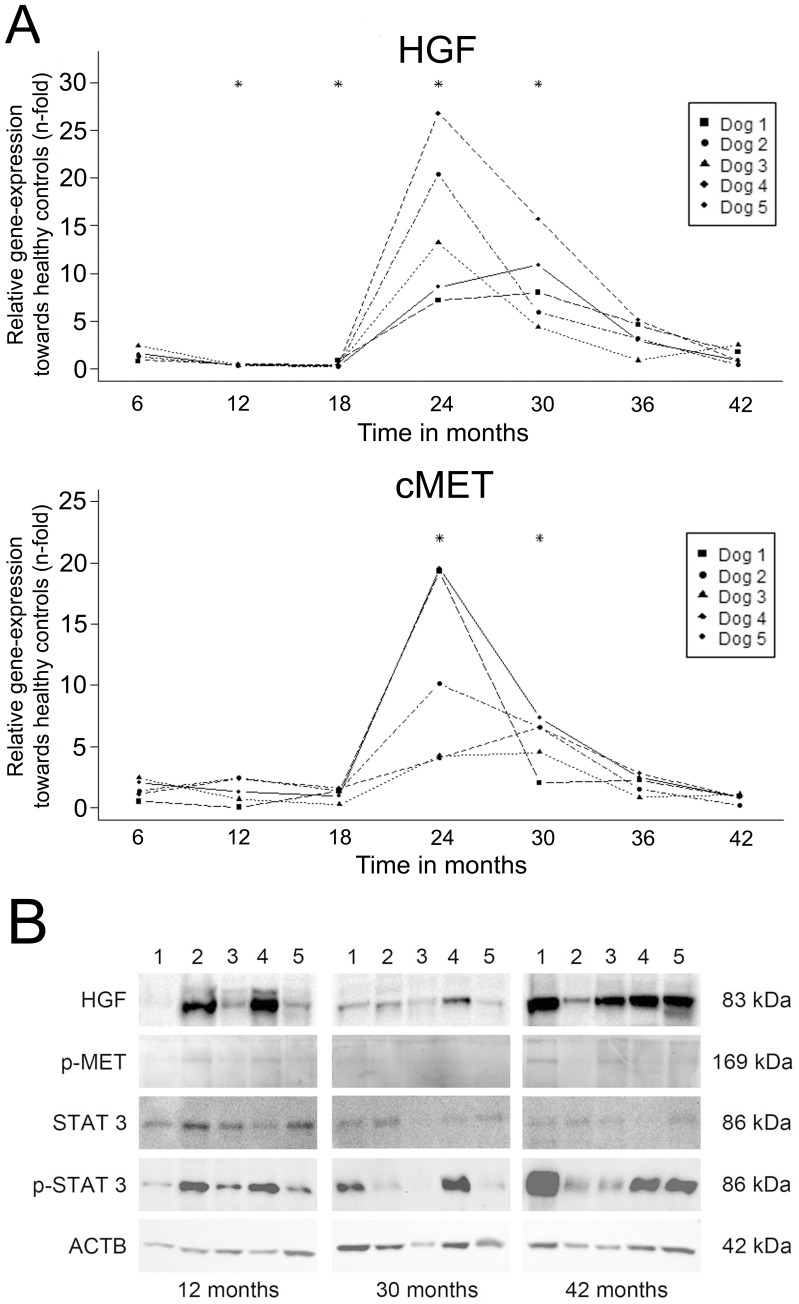
HGF pathway. (**A**) Gene-expression profiling. Interaction plots of Q-PCR data of important mediators of regeneration in a time-dependent copper-induced hepatitis in five COMMD1-deficient dogs. Q-PCR results were normalized against the expression of six control dogs (one to three years of age). Linear mixed-effect modeling was used; * significant difference corrected for multiple testing. (B) Western blot analysis on the activation HGF-signalling (HGF, phosphorylated c-Met, (phosphorylated) STAT3) of five COMMD1-deficient dogs at 12, 30, and 42 months. β-actin (ACTB) served as loading control.

The mRNA levels of TGF-β1 and both its receptors increased significantly starting at 24 months and decreased gradually afterwards (Figure 2A). The increase in relative mRNA levels of HGF and its receptor c-MET was sharp and strong (15 and 11-fold, respectively) occurring at 24 months of age in all dogs (Figure 3A). These levels decreased gradually over time and returned to basal levels at 36 months of age.

### Western Blot Analysis

Western blots were performed at the time points 12 (prior to elevation of mRNA levels of TGF-β1, HGF and their respective receptor), 30 (elevated mRNA levels), and 42 (mRNA levels returned to basal levels) months of age. Smad2/3 was detectable at all time-points. Phosphorylated Smad2 was present in the COMMD1-deficient dogs at all time-points (Figure 2B). HGF was variably present at all measured time-points in COMMD1-deficient dogs. Phosphorylated c-MET was increased age-dependently as was phosphorylated STAT3 (Figure 3B).

## Discussion

We evaluated COMMD1-deficient dogs as a possible large animal model for chronic hepatitis, in a 42 month lasting longitudinal study. A clear sequence of events occurred and explains in molecular terms the histological finding: copper accumulation, followed by hepatocellular damage, increased plasma ALT activity, hepatitis, HSC activation and fibrosis and further progression towards centro-central bridging fibrosis and cirrhosis. At the latest stages increased HGF proteins and activated STAT3 proteins were present.

Free copper is highly toxic due to its ability to generate hydroxyl radicals and it has been demonstrated that hepatocellular apoptosis is triggered by copper-induced cell damage [24], [25], [26]. Signs of regeneration appeared when the inflammatory process had progressed. This was mainly associated with hepatocytic regeneration shown by Ki67 immunostaining. LPC activation, reflected by non-cholangiocytic K19 immunostaining, was also present. In acute hepatitis LPCs differentiate through intermediate hepatocytes into hepatocytes within one week after the insult [27]. The fact that no intermediate hepatocytes were seen near the activated LPCs in the COMMD1-deficient dogs indicates a minor contribution of these cells to liver regeneration in this stage of hepatitis. The slow onset of disease and regenerative capacity of hepatocytes may explain that the endogenous LPCs do not contribute to the regeneration process in this model. One important multifunctional cytokine during liver regeneration is HGF [9], [10]. In this study, mRNA-expressions of HGF and its receptor c-MET peaked at 24 months of age. This coincided with hepatic stellate cell activation (α-SMA), and the expression of TGF-β1 and its receptors. Although HGF and c-MET expression were temporarily present, HGF, c-MET activation, and the down-stream time-dependent increase in phospho-STAT3 indicated a continuous activation of the regenerative pathway. High HGF protein levels at 42 months, most likely from an extra–hepatic source, stimulated STAT3 activation but cannot rule out increased inflammatory signaling via *e.g.* the IL-6 receptor towards STAT3 phosphorylation.

TGF-β1 signalling components (at mRNA and activated protein level) increased transiently from 24 months and coincided expectedly with an increased fibrosis (reticulin staining). The presence of pSMAD2/3 already at 12 months remains unexplained.

Until now COMMD1-deficient Bedlington terriers have been the best described dog model for hepatic copper toxicosis. Copper storage in COMMD1-deficient dogs causes chronic hepatitis, fibrosis and cirrhosis causing hepatic failure, portal hypertension, ascites, portosystemic collateral circulation, and hepatic encephalopathy. Therewith all features of chronic hepatitis and cirrhosis, not only copper-associated, are displayed. This experimentally reproducible autosomal recessive genetic disease may thus serve as an appropriate large animal model for chronic hepatitis and cirrhosis in man. Our study adds to the understanding of the sequence of pathophysiological events occurring in copper-associated hepatitis in COMMD1-deficient dogs and agrees with a semi-longitudinal study performed in *ATP7B^−/−^* mice [28]. COMMD1 mutations have so far not been associated with unexplained copper storage diseases in man [29], [30]. Although COMMD1 function remains puzzling [31], the major effects of its absence in the dog model seem to be impaired ATP7B-mediated copper export from the hepatocytes [22]. The COMMD1-deficient dog model is thus not the exact copy of Wilson’s disease, but shows all relevant features of human chronic hepatitis. This is the first well-defined large animal model of chronic hepatitis. We anticipate that it may provide an important instrument for the evaluation of future regenerative and anti-fibrotic therapies in a preclinical model and helps to close the gap between commonly used rodent models and human pathology.

In December 2011 a PlosOne paper described copper accumulation in liver-specific COMMD1 KO mouse [32] and confirmed the siRNA-based conclusion that COMMD1 plays an essential role in hepatic copper homeostasis [20], [33]. Surprisingly, these mice did not develop an obvious liver pathology under excessive copper diet. This clearly underscores the importance of this canine model as a long-term model for (therapeutic interventions into) chronic hepatitis.

## Materials and Methods

### Animals

Five new born COMMD1-deficient dogs with confirmed COMMD1 status (two males, three females) were used for longitudinal follow-up [34]. Dogs were examined every six months until the age of 42 months. At each occasion the clinical symptoms were scored, physical and blood examinations were performed, and liver biopsies were taken. Dogs were housed individually and received normal non-copper restricted commercial dog food (Noblesse, Purina), once a day and water *ad libitum*. The procedures were approved by Utrecht University’s Ethical Committee, as required under Dutch legislation (ID 2007.III.06.080).

### Blood Examinations

Serum alanine aminotransferase (ALT), alkaline phosphatase (AP), bile acids, and albumin were determined using a DXC-600 Beckman (Beckman Coulter, Woerden, the Netherlands). Reference ranges for ALT, AP, bile acids, and albumin are 0–54 U/l, 0–73 U/l, 0–10 µmol/l, and 26–37 g/L, respectively.

### Liver Tissue Sampling and Histopathology

Liver biopsies (five per dog per time-point) were obtained using a 14G Menghini needle [35]. Two biopsies were formalin-fixed and paraffin-embedded, and 4 µm thick paraffin sections were stained with haematoxylin and eosin (H&E), rubeanic acid (RA) for copper quantification, reticulin (Gordon and Sweet), and Sirius red. Hepatitis and fibrosis scoring were performed by one board-certified veterinary pathologist (TvdI) according to the WSAVA classification [36]. Copper accumulation (RA) was evaluated semi-quantitatively using a scale from 0 to 5 as previously described [37], and localization within the liver lobule was assessed. Three biopsies were fixed in RNAlater (Ambion, Austin, TX, USA) for a maximum of 24 hours and stored at −70°C.

### Immunohistochemistry

Alpha-smooth muscle actin (α-SMA), activated caspase-3 (Casp3), proliferation marker Ki67, Keratin 7 (K7), and Keratin 19 (K19) were stained as described elsewhere with slight modifications [5], [38]. The antibodies used for IHC are described in Table S1. Three µm sections were mounted on poly-L lysine coated slides and stored for a maximum of 48 hours at room temperature (RT). Sections were deparaffinised followed by antigen retrieval (AR). Enzymatic AR was performed with proteinase K (DAKO, Glostrup, Danmark) for 40 minutes (K7) or 15 minutes (K19). AR for Ki67 was achieved in 10 mM citric acid buffer (pH 6) for 15 minutes in a microwave. Activated casp3 and α-SMA staining needed no AR. Endogenous peroxidase and unspecific binding were blocked in 0.3% H_2_O_2_ and 10% non-immune goat serum for 30 min, respectively. After application of the primary antibody over-night (Casp3 and K7) or 1 h at RT (α-SMA, Ki67, and K19), slides were washed in PBS and incubated with the appropriate secondary antibody (Envision (DAKO) HRP-labeled polymer, anti-mouse or -rabbit) for 45 minutes at RT. After washing staining was detected using 3-3′-diaminobenzidine and counterstained with 10% Mayer’s haematoxylin.

α-SMA grading was performed as follows; 0: no staining of HSCs; 1: a few positive HSCs; 2: diffuse staining of HSCs; 3: mild increased staining of HSCs; 4: marked increased staining of HSCs. Results were compared with normal aged matched controls. The quantity of liver tissues from two needle biopsies was equal at all time points and in all animals; therefore both Ki67 and Casp3 were expressed as the number of positive hepatocytes per slide. K19 and K7 were graded as the number of positive cells in the limiting plate per portal area, either solitary or as small groups.

### RNA Isolation and Quantitative RT-PCR (q-PCR)

RNA was isolated from RNAlater (Ambion, Austin, TX, USA) fixed biopsies, using Qiagen RNeasy Mini Kit (Qiagen, Leusden, the Netherlands). RNA quality was analysed with the Agilent BioAnalyzer 2100 (Agilent, Palo Alto, CA, USA). A SYBR green I-based Q-PCR was performed on TFG-β1, TGF-β receptor type I (TGF-βR1), TGF-β receptor type II (TGF-βR2), hepatocyte growth factor (HGF), and c-MET protooncogene (c-MET) (Table S2) [15]. Normalisation was performed using four reference-genes (glyceraldehyde-3-phosphate dehydrogenase (GAPDH), hypoxanthine phosphoribosyl transferase (HPRT), ribosomal protein S5 (RPS5) and S19 (RPS19)) [39]. Q-PCR results were related to the expression of six clinically and histologically determined healthy control dogs (one to three years of age). Minus RT and water controls remained negative.

### Western Blot Analysis

Liver biopsies were lysed in RIPA buffer containing 50 mM Tris–HCl, 150 mM NaCl, 1% NP-40, 0.25% sodium deoxycholate, 1 mM EDTA, 1 mM sodium orthovanadate, 1 µg/ml aprotinin, and 1 mM PMSF. Extracted protein was quantified using a Lowry based assay (DC Protein Assay, Bio-Rad, Veenendaal, the Netherlands). To detect HGF, phospho-c-MET, (phospho-)STAT3 and (phospho-)Smad2/3 protein levels, 7.5 µg protein was denatured for 3 minutes at 95°C, subsequently run on a Criterion Tris-HCl polyacrylamide gel (Bio-Rad), transferred to Hybond-C nitrocellulose membranes (Amersham Biosciences, Roosendaal, the Netherlands) and blocked with the ECL Advance Western Blotting Detection Kit (Amersham, Little Chalfont, UK) or with non-fat dry milk (HGF). The blots were incubated overnight at 4°C with the appropriate primary antibodies (Table S3) and subsequently incubated with HRP-conjugated anti-mouse antibodies (R&D Systems Europe, Abingdon, UK; dilution 1∶20,000) or HRP-conjugated anti-rabbit antibodies (Santa Cruz Biotechnology, Santa Cruz, CA, USA; dilution 1∶20,000). All secondary antibodies were incubated for 1 hour at RT. Luminescence induced by the ECL Advanced Western Blotting Detection Kit (Amersham) was measured with a ChemiDoc XRS Imager (BioRad) and analyzed with provided software (Quantity-One 4.6.5).

### Statistical Analysis

Statistical analysis was performed in R (version 2.11.1) [40]. Q-PCR data and results of blood examination were analyzed using linear mixed-effect modeling with the R package “nlme”. When necessary, the outcome variables were transformed by their natural logarithm in order to fulfill the criteria of normality and constant variance. Restricted maximum likelihood method was used to estimate the best fitting model with a random intercept, a random slope, or both based on Akaike’s information criteria. Maximum likelihood was used to estimate the fixed effect of the time in months (included in the model as a factor). Residuals were checked for normality and constancy of variance. The Wilcoxon signed rank test with continuity correction was performed for comparisons of all time points with the reference time point (6 months) for differences in immunohistochemical data. Uncorrected p-values were reported in Table 2 and 3. P-values were considered significant, when the p-value times the number of comparisons was <0.05.

## Supporting Information

Table S1 Used antibodies in immunohistochemical experiments.
(DOC)

Table S2 Nucleotide Sequences of dog specific primers for Quantitative Real-Time PCR.
(DOC)

Table S3 Used antibodies in Western blot experiments.
(DOC)
