# Supplementary material for: COMMD1-Deficient Dogs Accumulate Copper in Hepatocytes and Provide a Good Model for Chronic Hepatitis and Fibrosis
Source: PLoS One. 2012 Aug 6;7(8):e42158. doi: 10.1371/journal.pone.0042158 (PMC3412840; doi:10.1371/journal.pone.0042158)
Supplement: Table S1 — Used antibodies in immunohistochemical experiments. (DOC) [file pone.0042158.s001.doc]

Favier et al. COMMD1 def dogs accumulate copper in hepatocytes and provide a good model for chronic hepatitis and fibrosis.

**Supplementary table 1**: Used antibodies in immunohistochemical experiments.

| **Name** | **Antibody** | **Dilution** | **Incubation** | **Positive control** | **Supplier** |
| --- | --- | --- | --- | --- | --- |
| α-SMA | mouse monoclonal | 1:200 in PBS | 60 minutes at RT | Hepatic artery smooth muscle | BioGenex, San Ramon, CA, USA |
| Caspase-3 | rabbit  polyclonal | 1:200 in PBS + 1% BSA | O/N at 4°C | Canine acute hepatitis | R&D Systems, Minneapolis, MN, USA |
| Ki67 | rabbit monoclonal | 1:50 in TBS | 30 minutes at RT | Canine duodenum | LabVision, Fremont, CA, USA |
| K19 | mouse monoclonal | 1:100 in TBS | 60 minutes at RT | Bile ducts | Novacostra, Newcastle upon Tyne, UK |
| K7 | mouse monoclonal | 1:25 in TBS + 1% BSA | O/N at 4°C | Bile ducts | Dako, Glostrup,  Denmark |

RT: room temperature; O/N: overnight
