# Supplementary material for: COMMD1-Deficient Dogs Accumulate Copper in Hepatocytes and Provide a Good Model for Chronic Hepatitis and Fibrosis
Source: PLoS One. 2012 Aug 6;7(8):e42158. doi: 10.1371/journal.pone.0042158 (PMC3412840; doi:10.1371/journal.pone.0042158)
Supplement: Table S2 — Nucleotide Sequences of dog specific primers for Quantitative Real-Time PCR. (DOC) [file pone.0042158.s002.doc]

Favier et al. COMMD1 def dogs accumulate copper in hepatocytes and provide a good model for chronic hepatitis and fibrosis.

**Supplementary table 2**: Nucleotide Sequences of dog specific primers for Quantitative Real-Time PCR

| **Gene** | **Primer sequence (5’🡪 3’)** | **Annealing temperature (°C)** | **Product size (bp)** | **Accession number** |
| --- | --- | --- | --- | --- |
| TGF-β1 | Forward CCAGGATCTGGGCTGGAAGTGGA | 66 | 113 | L34956 |
|  | Reverse CCAGGACCTTGCTGTACTGCGTGT |  |  |  |
| TGF-βR1 | Forward AGTCACCGAGACCACAGACAAAGT | 59 | 101 | AY455799 |
|  | Reverse TGAAGATGGTGCACAAACAAATGG |  |  |  |
| TGF-βR2 | Forward GACCTGCTGCCTGTGTGACTTTG | 61 | 116 | AY455800 |
|  | Reverse GGACTTCGGGAGCCATGTATCTTG |  |  |  |
| HGF | Forward AAAGGAGATGAGAAACGCAAACAG | 58 | 92 | BD105535 |
|  | Reverse GGCCTAGCAAGCTTCAGTAATACC |  |  |  |
| c-MET | Forward TGTGCTGTGAAATCCCTGAATAGAAAT | 59 | 112 | AB118945 |
|  | Reverse CCAAGAGTGAGAGTACGTTTGGATGAC |  |  |  |
| HPRT | Forward AGCTTGCTGGTGAAAAGGAC | 56 | 100 | L77488/9 |
|  | Reverse TTATAGTCAAGGGCATATCC |  |  |  |
| GAPDH | Forward TGTCCCCACCCCCAATGTATC | 58 | 100 | AB038240 |
|  | Reverse CTCCGATGCCTGCTTCACTACCTT |  |  |  |
| RPS5 | Forward TCACTGGTGAG/AACCCCCT | 62.5 | 141 | XM 533568 |
|  | Reverse CCTGATTCACACGGCGTAG |  |  |  |
| RPS19 | Forward CCTTCCTCAAAAAGTCTGGG | 61 | 95 | XM 533657 |
|  | Reverse GTTCTCATCGTAGGGAGCAAG |  |  |  |
