# Supplementary material for: COMMD1-Deficient Dogs Accumulate Copper in Hepatocytes and Provide a Good Model for Chronic Hepatitis and Fibrosis
Source: PLoS One. 2012 Aug 6;7(8):e42158. doi: 10.1371/journal.pone.0042158 (PMC3412840; doi:10.1371/journal.pone.0042158)
Supplement: Table S3 — Used antibodies in Western blot experiments. (DOC) [file pone.0042158.s003.doc]

Favier et al. COMMD1 def dogs accumulate copper in hepatocytes and provide a good model for chronic hepatitis and fibrosis.

**Supplementary table 3**: Used antibodies in Western blot experiments.

|  | Primary antibody | Dilution | Incubation | Product size (kDa) | Supplier |
| --- | --- | --- | --- | --- | --- |
| phospho-Smad2 (Ser465/467) | Rabbit polyclonal | 1:500 | O/N at 4°C | 52.5 | Cell Signaling Technology, Beverly, MA, USA |
| Smad 2/3 | Mouse monoclonal | 1:500 | O/N at 4°C | 52.5 | BD Transduction Laboratories, Franklin Lakes, NJ, USA |
| HGF | Rabbit polyclonal | 1:1,000 | O/N at 4°C | 83 | Abcam, Cambridge, UK |
| phospho-c-MET  (Y1230 + Y1234 + Y1235) | Rabbit polyclonal | 1:750 | O/N at 4°C | 169 | Abcam Cambridge, UK |
| phospho-STAT3 (Ser727) | Rabbit polyclonal | 1:1,000 | O/N at 4°C | 86 | Cell Signaling Technology, Beverly, MA, USA |
| STAT3 | Mouse monoclonal | 1:2,500 | O/N at 4°C | 86 | BD Transduction Laboratories, Franklin Lakes, NJ, USA |
| beta-actin (ACTB) | Mouse monoclonal | 1:2,000 | O/N at 4°C | 42 | Thermo Fisher Scientific, Fremont, CA, USA |

O/N: overnight
